# Examining the importance of local and global patterns for familiarity detection in soccer action sequences

**DOI:** 10.1177/03010066231223825

**Published:** 2024-01-10

**Authors:** Ed R. Hope, Keval Patel, James Feist, Oliver R. Runswick, Jamie S. North

**Affiliations:** 4589 Liverpool John Moores University, UK; Queens Park Rangers Football Club, UK; 2476 University of Chichester, UK; 34426 King's College London, UK; 62693 St. Mary's University, UK

**Keywords:** pattern recognition, local and global information, expertise, soccer

## Abstract

Pattern recognition is a defining characteristic of expertise across multiple domains. Given the dynamic interactions at local and global levels, team sports can provide a vehicle for investigating skilled pattern recognition. The aims of this study were to investigate whether global patterns could be recognised on the basis of localised relational information and if relations between certain display features were more important than others for successful pattern recognition. Elite (*n* = 20), skilled (*n* = 34) and less-skilled (*n* = 37) soccer players completed three recognition paradigms of stimuli presented in point-light format across three counterbalanced conditions: ‘whole-part’; ‘part-whole’; and ‘whole-whole’. ‘Whole’ clips represented a 11 vs. 11 soccer match and ‘part’ clips presented the same passages of play with only two central attacking players or two peripheral players shown. Elite players recognised significantly more accurately than the skilled and less-skilled groups. Participants were significantly more accurate in the ‘whole-whole’ condition compared to others, and recognised stimuli featuring the two central attacking players significantly more accurately than those featuring peripheral players. Findings provide evidence that elite players can encode localised relations and then extrapolate this information to recognise more global macro patterns.

Perceptual-cognitive skill has been defined as the ability to identify and acquire environmental information that is integrated with existing knowledge such that appropriate responses can be selected and executed (Marteniuk, 1976). The ability to encode, store and retrieve information has been proposed to enable skilled performers to anticipate events ahead of their happening and is especially important where performers operate under strict temporal constraints (for reviews, see Williams et al., 2011; Williams & Jackson, 2019). Perceptual-cognitive processes that encapsulate encoding, storage and retrieval have been linked to expert performance in military (see Endsley & Smith, 1996; Russo et al., 2005), medicine (see Abernethy et al., 2008; Sowden et al., 2000) and sport (see Williams, 2009).

One perceptual-cognitive skill that has consistently been shown to differentiate skilled from lesser-skilled performers across a variety of domains is the ability to perceive and recognise patterns within displays. The recognition paradigm involves presenting participants with a series of domain-specific stimuli in an initial viewing phase. Following a short break, participants then complete a recognition phase in which they are shown a further collection of stimuli – some of which were presented in the viewing phase and others that are novel – and are asked to respond to each stimulus as to whether they recognise it as having been presented in the earlier viewing phase or not. From its origins in chess (Goldin, 1978) to facial recognition research (e.g., Leder & Carbon, 2005), and sport (e.g., Williams et al., 2006), researchers have reported consistent findings. Specifically, higher skilled participants (variously classified as ‘experts’, ‘skilled’ or ‘elite’) demonstrate a recognition advantage (responding more accurately and/or quickly) over lesser skilled participants when responding to structured stimuli (i.e., those representative of typical game-based scenarios) but with this advantage being lost for unstructured stimuli (i.e., those in which display features are organised randomly or do not represent typical game-based scenarios).

More recently, researchers have progressed to investigating the processes that underpin pattern perception. For such research aims, invasion sports like soccer serve as useful vehicles given that they comprise of interactions between multiple display features (teammates, opponents, ball, playing field dimensions) which are dynamic, and hence require performers to make decisions under temporal constraints. In seeking to address *how* experts recognise patterns, Williams et al. (2006) asked skilled and less-skilled soccer players to complete pattern recognition paradigms under normal video-film conditions and when stimuli were converted to point-light sequences in the recognition phase. Point-light stimuli represented players as coloured dots against a black background (which retained playing field markings but removed all other visual information). Skilled soccer players demonstrated a recognition advantage over lesser-skilled in video-film *and* point-light conditions, providing initial evidence that skilled performers make use of relational information between display features (i.e., players and ball) to perceive and encode structure and meaning rather than isolated or superficial display features.

At a perceptual level, Dittrich's (1999) interactive encoding hypothesis has been used to explain pattern perception and recognition in environments or contexts comprising of multiple display features. Specifically, skilled performers initially employ ‘*bottom-up*’ low-level processes to extract motion information and temporal relationships between features, before engaging in high-level processing, where the stimulus presentation is matched with an internal semantic template employing higher-order ‘*top-down*’ processes (Didierjean & Marmeche, 2005; Gobet & Simon, 1996). Wong and Rogers (2007) propose a similar concept in their recognition of temporal patterns theory, whereby a pre-processing stage extracts only the essential information for pattern classification, before matching this information to a known template in memory. It is suggested that skill-level differences in pattern recognition arise because skilled performers have developed more complex and refined memory representations as a result of extended domain-specific practice which support efficient encoding, storage and retrieval of information (Ericsson & Kintsch, 1995). In contrast, and given their lack of domain-specific practice, novices develop less sophisticated memory representations, hence meaning cannot be extracted so readily, which impairs pattern recognition, and ultimately performance (Bilalić et al., 2010).

The findings from research that has employed point-light methods (see Williams et al., 2006) to manipulate the visual information available to participants has provided evidence that skilled players perceive and encode relational information between display features to successfully recognise patterns. Follow-up analyses employing visual search (North et al., 2009) and verbal report methods (North et al., 2011) indicated that the centrally located attacking display features and the patterns formed through their positions and movements between one another were especially important to successful recognition judgments. To more directly examine whether certain localised micro-relations (i.e., those between central attacking display features) were more important than others to pattern recognition judgments in soccer, North et al. (2017) employed a pattern recognition paradigm in which skilled and less-skilled players were initially presented with full-sided (i.e., 11 vs. 11) patterns of play shown as point-light stimuli in the viewing phase. In the subsequent recognition phase, stimuli were edited so as to only present localised micro-relations across three conditions; two peripheral players, two central attacking players, and two central attacking players as well as the player in possession of the ball. Findings showed that skilled players’ recognition accuracy was significantly higher in the conditions that preserved the relational information conveyed by central attacking features, with recognition accuracy improving further with the addition of the player in possession of the ball. It was concluded that within a global pattern, certain local patterns (i.e., peripheral players) may be redundant to the perception and recognition of the global pattern, whereas other local patterns are more important (i.e., the central attacking players and/or player in possession of the ball), when making pattern recognition judgments.

The proposal that localised patterns are essential to the recognition of a global pattern is consistent with attention and perceptual processes supporting familiarity judgments across other domains, including chess (e.g., see Bilalić et al., 2010) and face recognition (e.g., see Royer et al., 2015). In the latter context, and employing a similar method to North et al. (2017), Leder and Carbon (2005) manipulated both the *amount* and *order* of information presented in the viewing and recognition phases where either the ‘whole’ face or ‘parts’ of the face (nose, eyes and mouth in experiments 1 and 3, and just the eyes for experiments 2 and 4) were shown. Findings showed that participants were able to detect familiarity from ‘whole-whole’ and ‘whole-part’ presentation. However, when presented with ‘part’ stimuli in the viewing phase (i.e., nose, mouth and/or eyes) and then asked to recognise ‘whole’ stimuli in the recognition phase (i.e., the whole face), recognition accuracy was significantly impaired. From this, the authors concluded that it was necessary to encode the critical information within the context of the global pattern initially, for successful familiarity detection.

In another facial recognition study, Royer et al. (2015) restricted the amount of visual information presented to participants by employing a ‘*bubbles technique*’, which masks large areas of the face, but exposes other aspects through the medium of bubbles. When compared to novices, experts needed access to fewer facial features in the initial viewing phase, to make successful familiarity judgments in the subsequent recognition phase. In contrast to the work of Leder and Carbon (2005), who did not examine between group skill differences, the data suggested that experts were able to encode micro relations between key features (i.e., the eyes in the viewing condition) even when they were presented in the absence of the global pattern (i.e., the whole face), and that they could then extrapolate this localised relational information to recognise the global pattern in the subsequent recognition phase when the whole stimulus was presented. Moreover, Leder and Carbon (2005) did not seek to identify the relative importance of different types of facial features that may facilitate recognition.

The experimental approaches of Leder and Carbon (2005) and Royer et al. (2015) highlight a potential limitation with the methods used by North et al. (2017), who only employed a ‘whole-part’ order of presentation from the respective viewing to recognition phases. To this end, it is unclear whether pattern recognition in contexts involving multiple, dynamic, discrete display features (e.g., team invasion sports) can be successfully completed when ‘part’ or localised micro-relations between features are presented initially (i.e., in the viewing phase), whether successful pattern recognition can only be achieved if such micro-relations are initially encoded within the context of the global pattern (e.g., Leder & Carbon, 2005), or whether this is constrained by the nature of the ‘part’ information presented.

According to Wong and Rogers (2007) the fundamental challenge is for researchers to identify the minimal set of features which enable accurate pattern recognition. The study reported here extended upon the work of North et al. (2017) by introducing a novel ‘part-whole’ condition akin to that employed in the research by Leder and Carbon (2005) and Royer et al. (2015) examining facial recognition. The aim was to investigate whether global patterns could be recognised on the basis of localised relational information between select display features. We also sought to test whether relations between certain display features were more important than others for successful pattern recognition. In contrast to much of the research investigating skill-based differences in pattern recognition, we included multiple skill levels to establish if expertise rather than experience alone causes differences to emerge. Elite (professional), skilled (semi-professional) and less-skilled (recreational) participants completed three recognition paradigms of stimuli presented in point-light format. The premise here was to manipulate not only the type of information available, but also the order in which it was presented across three counterbalanced conditions (viewing → recognition phase): ‘whole-part’; ‘part-whole’; and ‘whole-whole’. Within the ‘part’ presentation, we manipulated the nature of the information that was displayed (hereon referred to as *Featured Players*), which resulted in either just the two central attacking players or peripheral players being shown.

Consistent with existing literature on pattern recognition (e.g., Williams et al., 2006), a main effect for expertise was hypothesised where more skilled participants would demonstrate superior overall recognition accuracy than their lesser skilled counterparts. In view of the research on facial recognition (Royer et al., 2015), a main effect of recognition paradigm condition was hypothesised, where recognition accuracy would be higher in the ‘whole-whole’ and ‘whole-part’ conditions when compared to the ‘part-whole’ condition. Additionally, this pattern of results was expected to be further pronounced in the more skilled players, resulting in a Skill Level×Recognition Paradigm interaction. When investigating the nature of information (*Featured Players*) displayed in the part conditions, a main effect for expertise was hypothesised, where more skilled participants would demonstrate superior recognition accuracy for stimuli in which the central attacking players were presented (North et al., 2009, 2017; Williams et al., 2012). In contrast, no skilled based differences/interactions were expected for the ‘part’ condition where only the peripheral players were shown (e.g., Royer et al., 2015).

## Method

### Participants

Using effect sizes from previous pattern recognition research (North et al., 2017), a priori power calculations suggested only a small sample size was required (*n* = 15) to detect the hypothesised Skill Level×Recognition Paradigm condition interaction. However, given criticism that experimental research is undermined by underpowered work with low sample sizes (Abt et al., 2020 cite a median sample size of *n* = 19), here we aimed to increase statistical power relative to previous pattern recognition research by recruiting a larger sample. To this end, 20 elite (*M* age = 26.4 years, SD = 5.23), 34 skilled (*M* age = 20.6 years, SD = 1.2) and 37 less-skilled (*M* age = 20.7 years, SD = 1.1) participants (all male) completed this study. Using the taxonomy put forward by Swann et al. (2015) for defining expertise, participants were considered elite if they had played, or were playing, professional soccer in the top 3 divisions of the English Football League (Championship, League 1, League 2). Participants were considered skilled if they had played, or were playing, soccer competitively at County (UK tier 11) level or higher (up to tier 6). Less-skilled participants were classified as such if they had never participated in soccer above recreational/Sunday league standard (matches typically played on Sunday, a lower standard of competition). Written informed consent was obtained from all participants and ethical approval was granted by the lead University's ethics board.

### Test Stimuli

**Figure 1.**
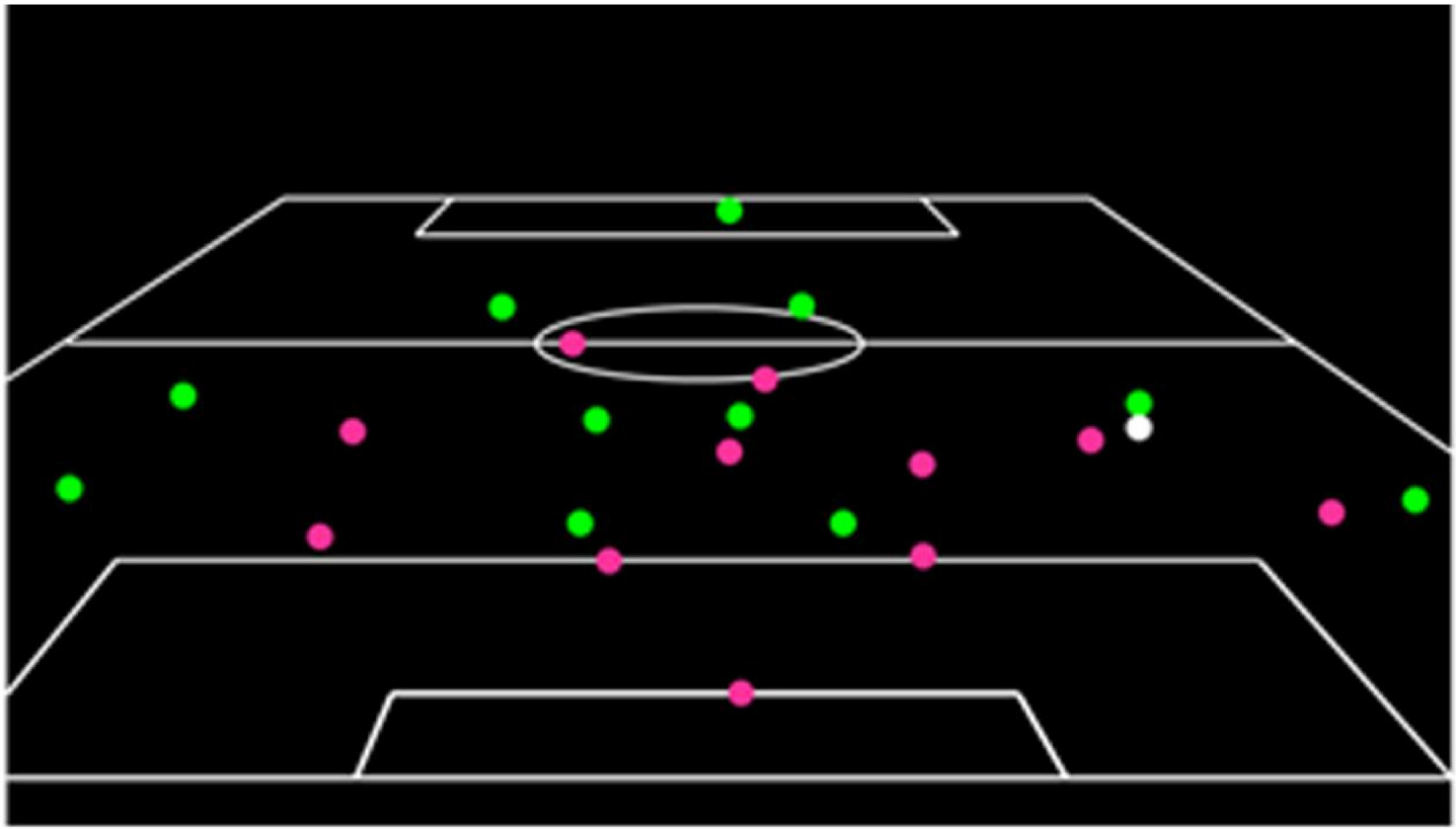
A frame from a point-light sequence of a soccer match in the ‘whole’ condition.

Stimuli were all structured soccer offensive sequences presented in point-light format (for an example, see Figure 1). All stimuli presented sequences that were filmed from a raised position, approximately 15 m behind the goal at a height of 9 m. Prior to their inclusion, the sequences (in normal video format) were independently assessed for structure by three coaches, all of whom held UEFA coaching licences, one holding the highest coaching qualification offered (UEFA Pro Licence). For each clip, coaches rated the degree of structure on a 10-point Likert-type scale (10 being a very highly structured sequence of play and 1 being highly unstructured). Only those clips with a mean rating of seven or higher were used in the study (as per the method previously employed by North et al., 2009, 2011 and Williams et al., 2006, 2012). Original film footage was converted into point-light format using the software package AnalysaSoccer2 (Liverpool John Moores University) which allowed .avi clips to be reconstructed by presenting points of light against a black background and then digitised. In point-light format all attacking players were displayed as green dots, defensive players were presented as pink dots and the ball as a white point of light (see Figure 1). Each sequence was 5 s in length, and would end when the player in possession was about to make an attacking pass. The inter-trial interval was 2-s. The stimuli were presented using an NEC video projector onto a 2.7 m×3.7 m screen. The test films were opened on a PC and displayed to participants through a Windows Media player (v10) at a rate of 25 frames per second. The stimuli for this study can be found at: https://osf.io/372x5/?view_only=c93a4624991e4d39b1164c81feaa2b8d

### Conditions

#### Order of Information Presentation

In total, participants completed three separate recognition paradigms that were counterbalanced within each of the three skill groups. The order and type of information presented across the viewing and recognition phases are outlined below.

*Recognition paradigm 1:*

**- Whole-whole:** presented 18 whole soccer clips (i.e., 11 offensive players, 11 defensive players and ball) in both the viewing and recognition phases.

*Recognition paradigm 2:*

**- Whole-part:** presented 18 whole soccer clips in the viewing phase, followed by 18-part clips in the recognition phase. The part clips were split evenly between the condition which presented just two peripheral players and the condition which presented just two central attacking players, with the order of these clips randomised (but kept consistent across participants).

*Recognition paradigm 3:*

**- Part-whole:** presented 18 part clips in the viewing phase, that were split evenly between the condition which presented just two peripheral players and the condition which presented just two central attacking players, with the order of these clips randomised (but kept consistent across participants). In the recognition phase, 18 whole clips were then presented.

For each of the three recognition paradigms, of the 18 stimuli that were shown in the recognition phase, 12 had been presented in the viewing phase and six were novel. For the ‘whole-part’ paradigm, previously shown and novel clips were split evenly between the Featured Players condition.

#### Featured Players

**Figure 2.**
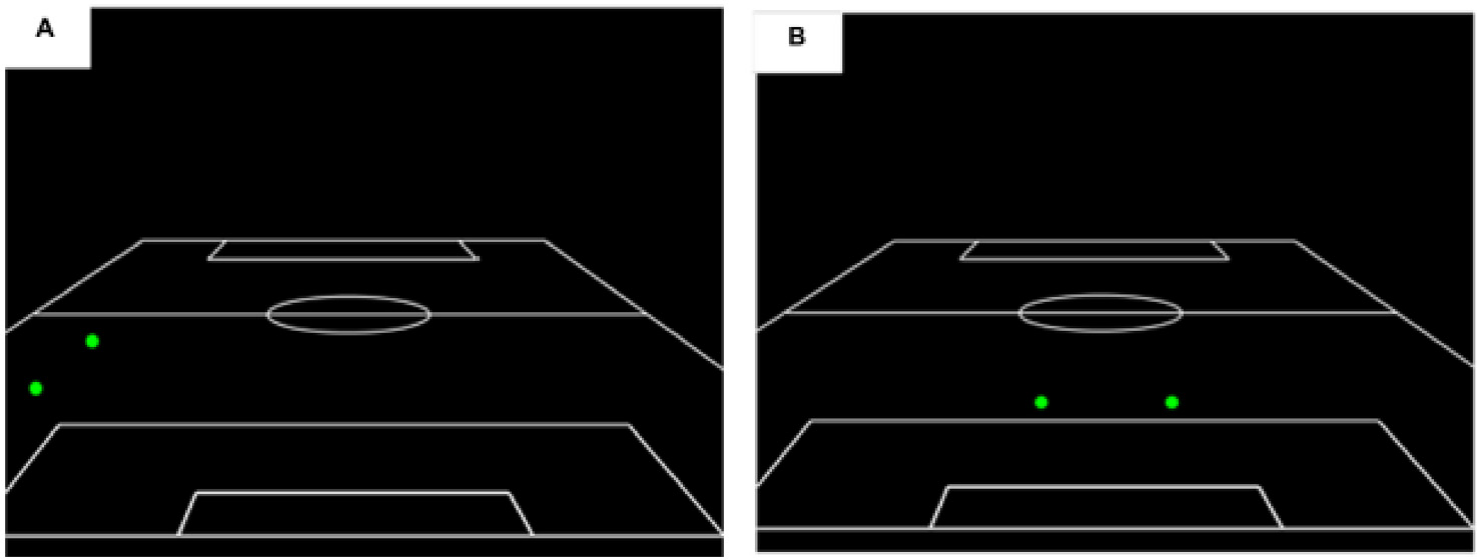
(A and B) A frame from a point-light sequence highlighting the two featured players conditions. *Note.* Both examples are for the ‘*Part*’ condition, with A depicting two peripheral players and B two central attacking players.

Across the viewing and recognition phases, stimuli were presented in either ‘whole’ or ‘part’ formats. For ‘whole’ clips all display features were presented (i.e., 11 offensive players, 11 defensive players, and the ball). However, for ‘part’ clips, the displays were edited to manipulate the visual information/featured players available to participants to form two conditions; two peripheral players from the team in possession (peripheral players condition), or two central attacking players from the team in possession (central attacking players condition). Example frames from the two ‘part’ conditions are shown in Figure 2A and B.

### Procedure

All participants sat in a chair 4 m from the projection screen, such that the image subtended a viewing angle of approximately 40°. Before participants received task instructions, they completed a sport history questionnaire to attain information on their playing experience. Participants were then told that they would be presented with a series of stimuli showing offensive soccer sequences that had been converted into point-light format. The concept of point-light stimuli was fully explained, with an example provided of a normal video clip and then its point-light equivalent.

Ahead of the viewing phase in each recognition paradigm, participants were informed that 18 5-s clips would be presented in total as either ‘whole’ (11 vs. 11 soccer game) or ‘part’ form (with only two select players shown). To familiarise participants with these display modes they were shown an example of a ‘whole’ and ‘part’ clip. When viewing the clips, participants were instructed to watch them as if they were a central defensive player but that no response was required during the viewing phase. Following the viewing phase, participants had a 10-min break before commencing the next part of the study; the recognition phase. For the recognition phase, participants were informed that they would be presented with a further 18 stimuli but that now they would be asked to provide a response following the presentation of each stimulus. Specifically, for the ‘whole-whole’ condition, they were asked to indicate whether they recognised each clip as having been presented in the preceding viewing phase (respond ‘yes’) or not (respond ‘no’). For the ‘whole-part’ and ‘part-whole’ paradigms, participants were instructed that some of the clips in the recognition phase were edited versions of clips that had been presented in the viewing phase, whereas others were edited versions of clips that had not been presented previously. So, in these recognition paradigms, participants were asked to respond ‘yes’ or ‘no’ as to whether each clip in the recognition paradigm was an edited version of one presented in the earlier viewing phase. For each of the three recognition paradigms, all participants were asked to watch each video clip in its entirety before responding, and if a participant missed a clip they were asked not to respond. There was a 60-min washout period between each recognition paradigm to reduce the potential effects of boredom and fatigue.

### Data Analysis

Recognition accuracy was determined by dividing the total number of correct recognition judgements by the total number of video clips presented and then multiplying this by 100 to calculate a percentage score for overall recognition performance and for each respective condition (*Recognition Paradigm* and *Featured Players*). To examine overall performance for *Skill Level* by *Recognition Paradigm*, a mixed design Analysis of Variance (ANOVA) was run, where the between-participant factor was *Skill Level* (elite vs. skilled vs. less skilled) and the within-participant factor *Recognition Paradigm* (‘whole-part’ vs. ‘part-whole’ vs. ‘whole-whole’).

To isolate the effects of the *order* in which the information was presented as well as the *type* of the information presented in the ‘part’ conditions (i.e., *Featured Players*), we removed the ‘whole-whole’ condition from our analyses. We then conducted a second mixed design ANOVA, where *Skill Level* was the between participants factor and there were two within participants factors; *Recognition Paradigm*, comparing performance between the ‘part-whole’ and ‘whole-part’ conditions, and *Featured Players* in the ‘part’ conditions, comparing performance between the two central offensive players and the two peripheral players. Partial eta squared values were calculated to provide measures of effect size for interactions and main effects and Cohen's *d* values were also calculated for comparisons between two means. All post-hoc tests were conducted using Bonferroni-corrected comparisons with the alpha level for statistical significance set at *p* < .05. The full data for this study can be found at https://osf.io/372x5/?view_only=c93a4624991e4d39b1164c81feaa2b8d

## Results

### Total Performance for *Skill Level* by *Recognition Paradigm* Condition

There was a main effect of *Skill Level* on recognition accuracy (F _2, 88 _= 52.093, *p* < .001, ŋ *_p_* ^2^ = 0.54). Post-hoc comparisons showed that elite players recognised more accurately than the skilled *p* < .001, *d* = 0.850) and less-skilled groups (*p* < .001, *d* = 1.05), respectively. There was no difference in recognition accuracy between the skilled and less-skilled groups (*p* = 0.121, *d* = 0.22) (Table 1).

**Table 1.** Mean recognition accuracy (%) across paradigm condition as a function of skill level.

Group/Condition	Whole-whole	Whole-part	Part-whole	Total
Elite	77.25 (8.80)	68.45 (5.54)	69.30 (12.53)	71.67 (10.06)
Skilled	63.00 (12.38)	55.56 (9.09)	51.00 (13.52)	56.52 (12.70)
Less-skilled	61.03 (13.17)	48.24 (10.53)	50.41 (12.31)	53.22 (13.20)
Combined	67.09 (11.45)	57.42 (8.36)	56.90 (13.12)	60.47 (9.84)

**Figure 3.**
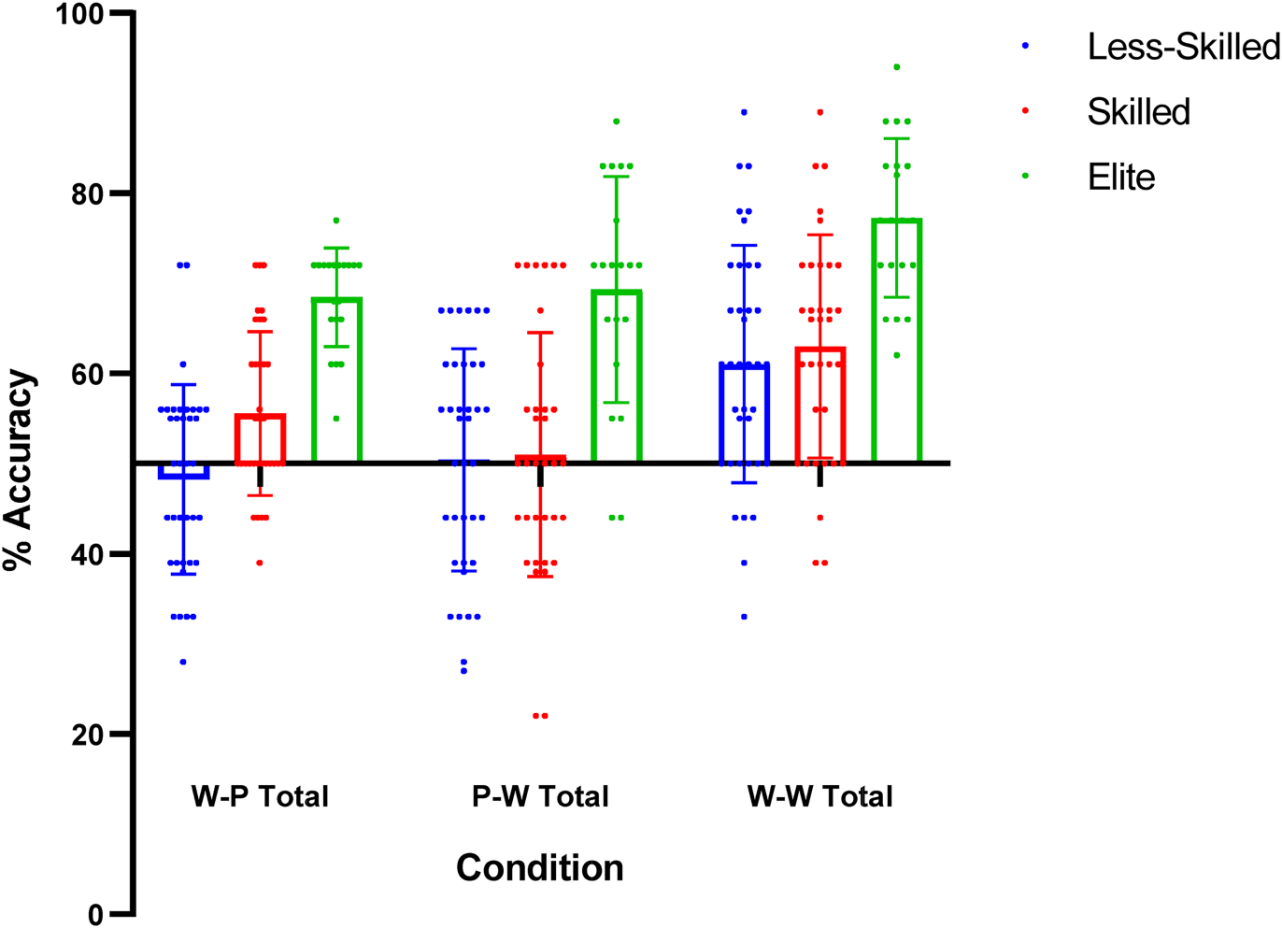
Recognition accuracy (%) scores across the paradigm conditions. *Note.* Whole-whole condition = W-W, whole-part condition = W-P and part-whole = P-W. Individual data points are presented alongside mean and standard deviation.

There was a main effect of *Recognition Paradigm* on recognition accuracy, (F _2, 176_ = 21.503, *p* < .001, ŋ *_p_* ^2^ = 0.196). Post-hoc comparisons revealed that participants were more accurate in the ‘whole-whole’ condition compared to the ‘whole-part’ (*p* < .001, *d* = 0.58), and the ‘part-whole’ conditions (*p *< .001, *d* = 0.61), respectively. There was no difference between ‘whole-part’ and ‘part-whole’ (*p* = 0.769, *d* = 0.03) conditions. There was also no *Skill Level* by *Recognition Paradigm* interaction (F _4, 176_ = 1.030, *p* = 0.393, ŋ *_p_* ^2^ = 0.023; Figure 3).

### Featured Players

**Figure 4.**
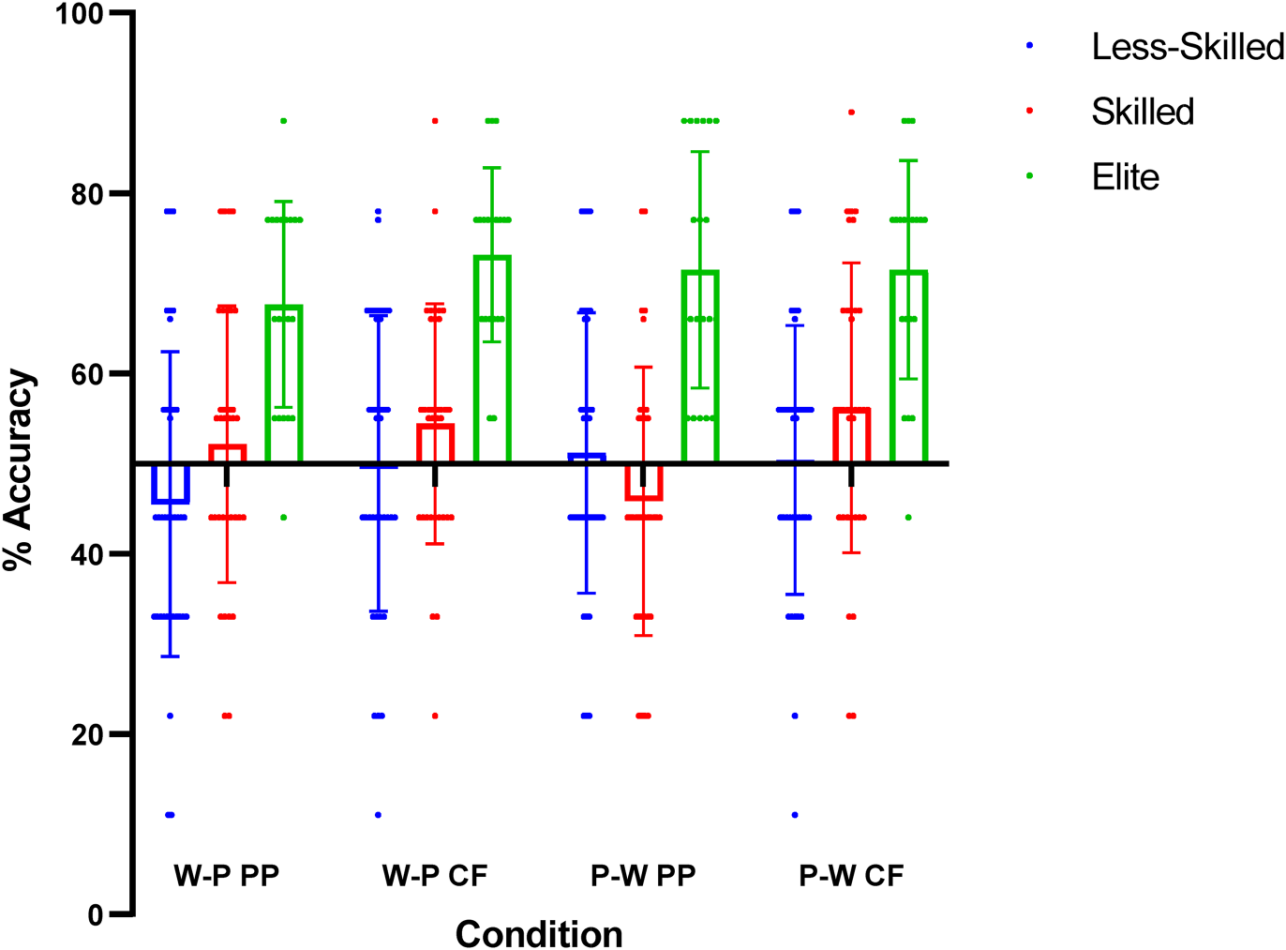
Recognition accuracy (%) scores for the featured players conditions. *Note.* Whole-part condition = W-P, and part-whole condition = P-W. Peripheral players condition = PP, and central attacking players condition = CF. Individual data points are presented alongside mean and standard deviation.

As per Figure 4, when considering the visual information presented in the ‘part’ conditions, there remained a main effect of *Skill Level* on recognition accuracy (F _2, 88 _= 57.043, *p* < .001, ŋ *_p_* ^2^ = 0.57). Post-hoc comparisons showed that elite players recognised more accurately than the skilled (*p < *.001, *d* = 0.92) and less-skilled (*p < *.001, *d* = 1.08) groups, respectively. There was no difference between the skilled and less-skilled groups (*p* = .345, *d* = 0.17). Similarly, there was no main effect of *Recognition Paradigm* on recognition accuracy, (F _1, 88_ = 0.161, *p* = .690, ŋ *_p_* ^2^ = 0.002) (Table 2).

**Table 2.** Recognition accuracy (%) as a function of order of information presentation and skill level for the featured players conditions.

Order	Condition	Group	Mean (SD)
Part-whole	Central forwards	Elite	71.50 (12.10)
		Skilled	56.17 (16.06)
		Less-skilled	50.41 (14.93)
	Peripheral players	Combined	59.36 (10.9)
		Elite	71.50 (13.11)
		Skilled	45.82 (14.89)
		Less-skilled	51.19 (15.54)
		Combined	56.17 (11.06)
Whole-part	Central forwards	Elite	73.15 (9.63)
		Skilled	54.44 (13.31)
		Less-skilled	50.03 (16.42)
		Combined	59.21 (12.27)
	Peripheral players	Elite	67.65 (11.44)
		Skilled	52.15 (15.34)
		Less-skilled	45.49 (16.91)
		Combined	55.10 (11.37)

There was, however, a main effect of *Featured Players* (F _1, 88_ = 5.977, *p* = .016, ŋ *_p_* ^2^ = 0.064). Stimuli featuring the two central attacking players were recognised more accurately than those featuring the peripheral players (*p* = .016, *d* = 0.26).

There was no *Skill Level*×*Recognition Paradigm* interaction (F _2, 88_ = 1.279, *p* = .283, ŋ *_p_* ^2^ = 0.028), *Skill Level×Featured Players* interaction (F _2, 88_ = 0.994, *p* = .374, ŋ *_p_* ^2^ = 0.022) or *Recognition Paradigm*×*Featured Players* interaction (F _1, 88_ = 0.72, *p* = .789, ŋ *_p_* ^2^ = 0.001). There was also no three-way interaction between *Skill Level*, *Recognition Paradigm* and *Featured Players* (F _2, 88_ = 1.923, *p* = .152, ŋ *_p_* ^2^ = 0.042).

## Discussion

In this study we aimed to further investigate whether skilled performers were able to recognise global patterns on the basis of localised relational information between select display features. Having hypothesised that skilled performers would be able to recognise global patterns through localised relational information (North et al., 2017; Williams et al., 2006), we also sought to test whether relations between certain display features were more important than others for successful pattern recognition. We found skill-based differences across all three recognition paradigms, with elite players being significantly more accurate in recognising stimuli than the skilled and less-skilled groups, respectively.

While we cannot confirm the exact causal mechanisms by which expertise effects were observed, given the nature of the stimuli employed, our findings lend support to the interactive encoding hypothesis proposed by Dittrich (1999). Specifically, skilled performers initially employ low-level processes to extract motion information as well as temporal relationships between features before engaging in high-level processing, where the stimulus presentation is matched with an internal semantic template to govern skilled familiarity judgements (Didierjean & Marmeche, 2005; Gobet & Simon, 1996). In view of the recognition of temporal patterns theory conceptualised by Wong and Rogers (2007), expertise effects in pattern recognition can arise because skilled performers have developed more complex and refined higher-order memory representations as a result of extended domain-specific practice which support efficient encoding, storage and retrieval of information (see Long-Term Working Memory Theory by Ericsson & Kintsch, 1995). This could explain the main effect for skill level throughout all three recognition paradigms in the present study, especially given the relative approach employed (Chi, 2006), and the three distinct skill levels examined with ‘*real*’ experts recruited (Swann et al., 2015). As predicted, there was a main effect of *Recognition Paradigm* on performance, with participants significantly more accurate in the ‘whole-whole’ condition than the ‘whole-part’ and ‘part-whole’ conditions. These findings support previous research, where superior familiarity judgments are observed when full-sided stimuli are presented in both the viewing and recognition phases, respectively (e.g., North et al., 2017; Williams et al., 2006; Williams & Davids, 1998). This finding is also consistent with research investigating facial recognition, where whole faces were recognised more easily than a collection of facial features presented separately, owing to a greater number of important configurations and holistic processing (Leder & Carbon, 2005). From a theoretical perspective, the encoding specificity principle (Tulving & Thomson, 1973) may help to explain findings, and the importance of maintaining specificity between encoding (i.e., viewing phase) and retrieval contexts (i.e., recognition phase), which facilitated recognition performance in the ‘whole-whole’ condition for all participants in the present study.

Consistent with the findings reported by North et al. (2017), the nature of information in the ‘part’ conditions affected recognition. A main effect of *Featured Players* was observed, where participants were more accurate in recognising stimuli that retained the positions and movements of central offensive features than stimuli that presented positions and movements of peripheral players, supporting the importance of these micro relations to pattern recognition. However, and contrary to our predictions and the work on facial recognition by Royer et al. (2015), there was no *Skill Level*×*Recognition Paradigm* interaction, as elite players showed no difference between the ‘part-whole’ and ‘whole-part’ paradigms. Further, there was no three-way interaction between *Skill Level*, *Recognition Paradigm* and *Featured Players*. Our findings therefore suggest that for the ‘part-whole’ recognition paradigm condition, experts were able to encode micro relations between key features in the initial viewing phase and then extrapolate this information in the subsequent recognition phase where the whole pattern was presented to successfully inform their familiarity judgments.

Similarly, and replicating the work of North et al. (2017), our findings suggest that for the ‘whole-part’ condition, experts were able to encode the key localised micro relations from the whole pattern and then accurately recognise this information in the subsequent recognition phase where only the central attacking players were presented. In view of the aforementioned encoding specificity principle (Tulving & Thomson, 1973) it appears that the micro relations initially presented maintained sufficient specificity between the encoding and retrieval contexts to facilitate successful recognition performance for the elite players. With regards to the other skill levels, there was also no main effect of *Recognition Paradigm* on recognition accuracy. Crucially, however, their performance was around chance level (skilled = 52.15%, less skilled = 49.28%) with no interaction effects observed across the conditions. This lends further support to conclusions drawn in previous research that experts can recognise global patterns through micro relations between key display features (i.e., those centrally located), whereas lesser-skilled players appear unable to do so (see North et al., 2017).

While our work is more conceptually driven, from an applied perspective the finding that experts can recognise global patterns having only previously been presented with localised relations between certain key display features, seemingly lends support to the growing popularity of small-sided games (SSGs) as a training method in sport to enhance physical, technical and tactical skills (Sarmento et al., 2018). More specifically, training in practice contexts where players are exposed to just two or three opponents could develop important perceptual-cognitive skills that transfer to a full-sided context. This seems particularly pertinent given pattern recognition has been consistently shown to be a defining characteristic of expertise in team-based sports (Abernethy et al., 2005; Gorman et al., 2011; Williams & Davids, 1995).

In this study, we garnered a considerably larger sample than in most previous research in this area. Further, we recruited an elite level group comprising professional soccer players, who demonstrated clear expertise effects relative to the lesser skilled groups. The lack of skill-based differences between these latter groups alongside the increased statistical power and enhanced group structure, suggests that you have to be a ‘*real*’ expert before skill level effects are observed. To this end, expertise studies in this field must endeavour to recruit highly skilled samples (Swann et al., 2015). Nevertheless, there are some limitations that are important to highlight. Specifically, future research may wish to employ more realistic video footage, such as first-person viewing perspectives (e.g., Roca et al., 2013) or immersive technology to increase both the action fidelity and functionality of the task, in order to elicit greater expert-novice differences (Stone et al., 2018; Travassos et al., 2013). Akin to previous research, but more of a challenge when increasing power, processing tracing measures such as verbal reports or visual search behaviour could also be employed to gain a greater understanding of the nature of information constraining familiarity judgements across the different recognition paradigms (North et al., 2009, 2011; Roca et al., 2011). To this end, and while we have explained our findings through the two-stage interactive encoding hypothesis proposed by Dittrich (1999), a more direct measure of the low-level processes employed to extract and encode motion information would help to broaden our understanding of the causal mechanisms underpinning the expertise effects observed. For example, we cannot discount how these may have resulted from superior lower-level memory structures (i.e., short-term memory) in the encoding and retrieval of information, rather than high-level processes (e.g., LTWM). Finally, future work may wish to undertake qualitative research on pattern recognition to garner richer information on this perceptual-cognitive skill from both a player and coach perspective, which may better inform training design, especially around the use of SSGs for tactical development in sport.

In this paper, we have extended understanding of perceptual processes informing pattern recognition in environments comprising of multiple dynamic features by manipulating both the *type* and *order* in which visual stimuli were presented. Specifically, and in line with previous research (e.g., North et al., 2017) central attacking players appear to be crucial features constraining pattern recognition for soccer action sequences. Additionally, and through our experimental design employing a ‘part-whole’ condition, we have provided novel findings to more directly evidence that elite players are able to encode localised relations between key features and then extrapolate this information to recognise more global macro patterns; whereas lesser-skilled players appeared unable to do so. Our findings have potentially important implications for practice design in developing pattern recognition expertise in team-based sports, albeit further research is needed to more directly investigate this concept.
